# Primary User Localization and Its Error Analysis in 5G Cognitive Radio Networks

**DOI:** 10.3390/s19092035

**Published:** 2019-04-30

**Authors:** Nasir Saeed, Haewoon Nam, Tareq Y. Al-Naffouri, Mohamed-Slim Alouini

**Affiliations:** Division of Electrical Engineering, Hanyang University, Ansan 15588, Korea; mr.nasir.saeed@ieee.org (N.S.); tareq.alnaffouri@kaust.edu.sa (T.Y.A.-N.); slim.alouini@kaust.edu.sa (M.-S.A.)

**Keywords:** primary users, secondary users, received signal strength, localization

## Abstract

It is crucial to estimate the location of primary users (PUs) for the development of cognitive radio networks (CRNs). Great efforts have been made in the past to develop localization algorithms with better accuracy but low computation. In CRNs, PUs do not cooperate with secondary users (SUs), which makes the localization task challenging. Due to this feature, received signal strength (RSS)-based PU localization techniques, such as centroid localization (CL) and multidimensional scaling (MDS), are the best candidates. However, most of the CL- and MDS-based PU localization methods consider omnidirectional wireless communication. Therefore, in this paper we propose a PU localization method which uses the RSS values at different sectors of the SU antenna, where a scoring strategy is applied to all the sectors to estimate the PU location. Two different scoring functions are proposed. Numerical results show that the proposed localization method is robust to PU locations and channel conditions. The proposed method is validated in terms of various network parameters, such as the number of SUs, beamwidth of the SU sectors, size of the grid, and placement of the PUs. Results show that increasing the number of SUs improve the localization accuracy due to an increased number of measurements. However, the localization accuracy degrades with an increase in the beamwidth of the SU sector because the faraway grid points also participate in the localization. The results are also compared with the conventional CL for PU localization. Compared with conventional CL, it offers a significant improvement in the performance.

## 1. Introduction

The radio spectrum is one of the most valuable resources in wireless communication systems. Therefore, to efficiently use the spectrum, dynamic spectrum access has been introduced by using cognitive radio networks (CRNs) [1,2,3,4]. The spectrum is available to unlicensed secondary users (SUs) when the licensed primary users (PUs) are not active or do not use the spectrum fully in time, frequency, and space domain [5]. Hence, the SUs utilize the available spectrum by sensing the spectrum in time, frequency, and space domain. The SUs are able to utilize the spectrum by using an overlay mechanism if it is in the coverage region of a PU; otherwise, it uses an underlay mechanism if it is outside of the PU coverage. In the past decade, research on spectrum sensing in CRNs has mainly focused on time- and frequency-based spectrum sensing, and few works exist on spatial spectrum sensing. However, the channel statistics between the PU and SU may not be perfect, and can lead to possible interference to the PU in time- and frequency-based approaches. Therefore, recently, there has been great interest in developing PU localization schemes where the location information of a PU can be of great benefit to provide spectrum-sensing in the spatial domain.

Although CRNs provide efficient use of the scarce RF spectrum, it introduces security issues, such as primary user emulation (PUE) attacks. In PUE, an attacker emulates the PU signals to deter the SUs from accessing the unlicensed spectrum. Various approaches were developed by the researchers to cope with PUE attacks [6,7,8,9,10]. For example, a localization-based approach was used in [6] to detect the PUE attack in the unused TV spectrum. Similarly, an analytical model was used in [7] to find the probability of detection for PUE in energy-based spectrum sensing. Moreover, a non-parametric and passive classification method was used in [8] to detect the emulated signal from a malicious user. Cooperative and non-cooperative detection schemes were proposed in [9] that are based on tapping the channel power between the transmitter and receiver. Morevoer, a multipath delay-based PUE detection mechanism was analyzed in [10]. Other techniques for PUE detection include the time difference of arrival (TDoA) approach to estimate the location of the PU transmitter [11]. Although the TDoA approach provides better accuracy, it requires tight synchronization between the transmitter and the receiver. Similarly, the angle-received signal-strength-based approaches were used in [12,13] to detect the PUE attack. Recently, a PUE detection mechanism for non-stationary PU was proposed in [14] by using the Kalman filter.

Besides the PUE detection in CRNs, localization of a PU in CRNs enable various applications, such as radio resource management in the space domain, efficient routing between the SUs without interfering the PUs, and reliable spectrum sensing [15]. There has been a great deal of attention in the past decade to develop localization techniques due to the increasing demand for location-based services. There are a number of applications which require location-based services (LBS), such as autonomous cars, emergency services, and public safety applications [16]. As the wireless communication networks have evolved from 2G to 5G, localization methods have also been improved significantly to meet the standards. The localization accuracy of 550 m was achieved in 2G wireless communication systems, while in 4G systems, the accuracy has been improved to tens of meters due to the time difference of arrival ranging. Moreover, the localization accuracy can be further improved to 10 m by using the assisted global navigation systems [17]. However, it is expected that the localization accuracy of 5G wireless communication systems will be in the range of 1 m to 10 m for outdoor scenarios, while the indoor localization accuracy is expected to be less than a meter [18]. Localization methods for cognitive radio networks (CRNs) can be broadly classified into range-free and range-based methods. Range-free methods do not require actual ranging between the PU and the SUs, and depend only on the connectivity information. The range-free localization methods are simple but have low accuracy. On the contrary, localization methods based on ranging are more accurate but have more complexity and cost. Hybrid range-based and range-free localization methods have also been proposed in [15,19,20] for PU localization in CRNs.

The features of 5G networks, such as ultra-dense networks, wide bandwidth, smart antennas, and device-to-device communication will provide high capacity, low delay, and energy-efficient communication. 5G networks are expected to consist of a huge number of heterogamous devices which may use the licensed frequency band or the unlicensed frequency band. Therefore, the 5G networks can benefit from the CRN concept to significantly increase the spectrum efficiency. However, it will also introduce new challenges, such as optimization of the coexistence of various systems and technologies. Furthermore, the wide bandwidth of the 5G networks is likely to be implemented by mmWaves, which requires small-sized directional antennas. Also, the nodes are expected to have smart antennas with the capabilities of multiple input multiple output (MIMO) techniques. Thus, these small-sized directional antennas can provide angle information of the arrived signal at the receiver which can be helpful for localization [21].

Motivation for this paper was how the use of directional sectorized antennas is becoming more widespread for different applications. These directional antennas can achieve longer transmission distance and better performance, since the transmission beam is focused to a specific direction. Therefore, such features of directional antennas should be considered for localization as well where most of the localization research is focused on omnidirectional wireless communication. Even though the cells are sectorized in cellular networks, for localization purposes, it is assumed that the antennas are omnidirectional [22].

The remainder of the paper is organized as follows. Section 2 presents related studies. In Section 3 and Section 4, we present the system model and the proposed PU localization method, respectively. In Section 5, we analyze the localization error of the proposed method. Section 6 presents the numerical results to evaluate the performance of the PU localization method, and we compare the results with CL. Section 7 concludes the work, with a few remarks.

## 2. Related Studies

Based on the transmission capabilities of PUs and SUs, the research on PU localization in CRNs can be broadly categorized into the two following categories:

- Omni-directional: There has been extensive research focusing on omnidirectional CRNs localization. Such omnidirectional localization methods estimate the location of a PU by using fingerprinting, trilateration, and multidimensional scaling-based estimation methods [15,19,20,23,24,25,26]. The simplest PU localization method among all these methods is centroid localization (CL) [23], which averages out the coordinates of the SUs to estimate the PU location. CL is a very simple and range-free approach, but due to its center-biased nature, it cannot achieve high accuracy. Therefore, the CL has been modified to weighted centroid localization (WCL) in [24], such that the localization performance is improved by using the weighting strategies. The weighting strategies for WCL are based on the received power, where the accurate measurements are given more weight, and vice versa. Another variant of WCL was proposed in [27] which tries to find a good match between the measured power and the nearest value to that power in a database by using mean square error. In [15,19,20,26], multidimensional scaling-based network localization methods were used to estimate the location of SUs and PUs, where both range-based and range-free schemes are utilized. All of the aforementioned localization methods consider how both PUs and SUs are using omnidirectional antennas. However, a number of current applications require the use of directional antennas to achieve better performance and a long transmission distance.
- Directional: Recently the work in [28,29,30,31] have shown that the use of directional antennas certainly reduces the localization error. In [31] the authors have investigated the impact of directional antennas on the localization performance of sectorized cellular networks. A directional of arrival (DoA)-based localization method for PU localization has been proposed in [29,30] where the SUs are equipped with multiple antennas. DoA measurements are estimated by using the multiple signal classification (MUSIC) method, and the Stansfield estimator is used to estimate the location of a PU. A joint DoA and received signal strength (RSS)-based PU localization method has been proposed in [28] which improves the localization performance, as compared to DoA-only and RSS-only methods.

In this paper, we investigate the problem of PU localization in CRNs in the presence of sectorized directional antennas at the SUs. We also analyze the error performance of the proposed method for two-dimensional PU localization. Moreover, the root mean square performance (RMSE) of the proposed method is compared to the conventional CL, where the results show that the proposed method is not center-biased and robust.

## 3. System Model

**Figure 1:**
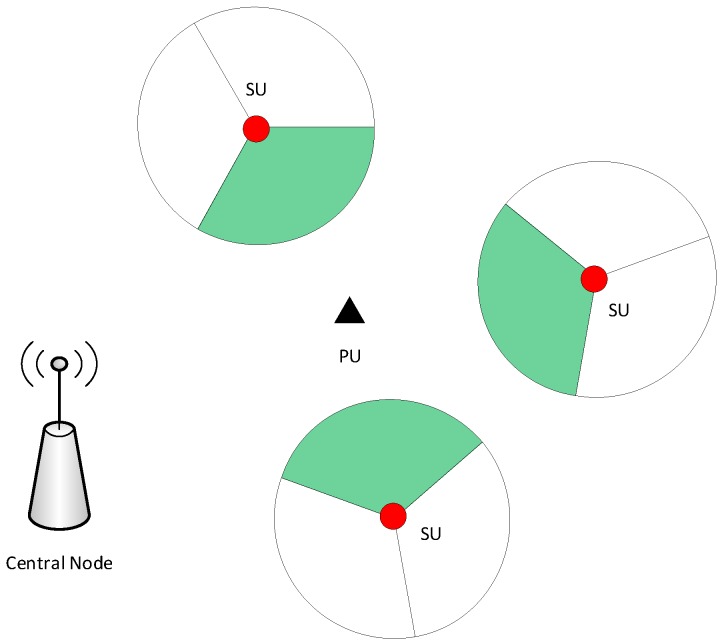
System model of the CRN: the black triangle represents the PU location, and the red circles represent SUs. Each SU has three sector antennas, where the green sectors are able to detect the PU’s presence.

We considered a two-dimensional CRN setup, which consists of *N* SUs and a PU deployed in a square area of $L\times L\;m^{2}$, as shown in Figure 1. The two-dimensional location of *i*-th SU is denoted by $l_{i}=\left\{x_{si},y_{si}\right\}$ and the location of the PU is denoted by $l_{p}=\left\{x_{p},y_{p}\right\}$. A central unit was considered, which collects the PU presence and absence information from the SU and estimates the location of the PU. This setup can be used to model different cellular network architectures, ranging from macrocells to femtocells [32,33]. The transmission of the PU is assumed to be omnidirectional, while the SUs are equipped with *M*-sector antennas where the RSS at each sector is characterized by the angular sector and path loss model. Based on the aforementioned network setup, the power received from the PU at the *m*-th sector of the *i*-th SU is given by

(1) $$P_{im}=\frac{P_{t}G_{t}G_{R,M}\left(\theta_{im},\phi_{im}\right)}{\rho (d)}$$

where $P_{t}$ is the transmit power of PU, $G_{t}$ is the transmit antenna gain, ρ(d) is the path loss at distance *d*, and $G_{R,M}$ is the receive antenna gain, which depends on $\theta_{im}$ (boresight angle) and $\phi_{im}$ (angle of reception). Based the angle of reception, the receive antenna gain $G_{R,M}\left(\theta_{im},\phi_{im}\right)$ is defined as

(2) $$G_{R,M}\left(\theta_{im},\phi_{im}\right)=\left\{\begin{array}{cc} G_{R,M} & \mathrm{if}\;\phi_{im}\in \left[-\phi_{0},\phi_{0}\right]\\ 0 & \mathrm{Otherwise} \end{array}\right.$$

where $\left[-\phi_{0},\phi_{0}\right]$ is the width of the sector. We assumed that the SUs were randomly distributed with a random orientation, where the propagation of signals was characterized by the distance-dependent path loss model. Therefore, the path loss from the PU at the *i*-th SU is given as

(3) $$\rho \left(d\right)=\rho \left(d_{0}\right)+10\eta \log_{10}\left(\frac{d}{d_{0}}\right)+S_{i}$$

where $\rho \left(d_{0}\right)=20\log_{10}\left(\frac{4\pi d_{0}}{\lambda }\right)$ is the path loss in dBs at a reference distance of $d_{0}$. η is the path loss exponent, λ is the wavelength, and $S_{i}$ is the shadowing effect which follows a zero mean normal distribution. It is assumed that the shadowing effect is independent across each SU.

## 4. Proposed PU Localization Method

**Figure 2:**
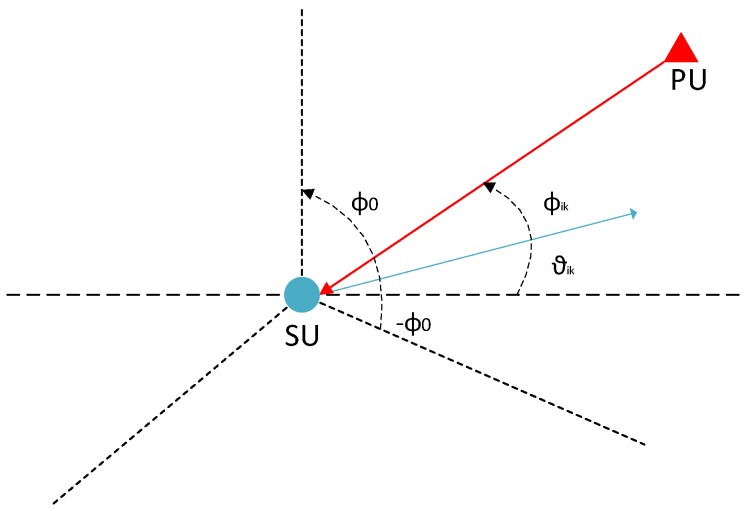
Illustration of finding the direction of a PU signal.

We considered a square region with a side length of *L* m, and divided the whole area into A×A grids, where the resolution of each grid is L/A m. The SUs are uniformly distributed, where each SU has three-sector antennas with a beamwidth of $\frac{2\pi }{3}$. The orientation of each SU is random and follows a uniform distribution. The proposed localization method is summarized as follows:

- In CRNs, the SUs carry out spectrum sensing to find out the existence or non-existence of PU signals in a specific channel. If the channel is idle, the signal received by the SUs is typically noise, but if the channel is busy, then the received signal consists of both the PU signal and the noise. An energy detection model is usually used in such a scenario to decide the presence or absence of the PU signal. The energy detection model consists of a binary testing hypothesis, with $H_{0}$ and $H_{1}$ for the PU’s absence and presence, respectively. The binary hypothesis for the detection of a PU is defined as [34]
  (4) $$H_{0}:y=\sum_{k=1}^{h}w\left(k\right),$$
  and
  (5) $$H_{1}:y=\sum_{k=1}^{h}\left\{s\left(k\right)+w\left(k\right)\right\},$$
  where *y* is the received signal, w(k) is the additive white Gaussian noise (AWGN) with zero mean and $\sigma_{w}^{2}$ variance, and s(k) is the PU signal with zero mean and $\sigma_{s}^{2}$ variance. Mainly, the energy detection-based PU detection is characterized by the probability of detection ($P_{d}$) and the probability of false alarm ($P_{f}$). The test statistics for PU detection is a random variable with a probability distribution function (PDF) of the chi-square distribution. If the number of observations *h* is large, the PDF of the test statistics can be approximated by the Gaussian distribution—that is:
  (6) $$H_{0}∼N\left(h\sigma_{w}^{2},2h\sigma_{w}^{4}\right),$$
  (7) $$H_{1}∼N\left(h\left(\sigma_{s}^{2}+\sigma_{w}^{2}\right),2h{\left(\sigma_{s}^{2}+\sigma_{w}^{2}\right)}^{2}\right).$$
  Using the principle of constant false alarm rate (CFAR) [35], $P_{f}$ and $P_{d}$ are given as:
  (8) $$P_{f}=p\left(y>\gamma |H_{0}\right)=Q\left(\frac{\gamma -h\sigma_{w}^{2}}{\sigma_{w}^{2}\sqrt{2h}}\right)$$
  and
  (9) $$P_{d}=p\left(y>\gamma |H_{1}\right)=Q\left(\frac{\gamma -h(\sigma_{s}^{2}+\sigma_{w}^{2})}{h\left(\sigma_{s}^{2}+\sigma_{w}^{2}\right)\sqrt{2h}}\right),$$
  respectively, where γ is the threshold for decision, and Q(·) represents the *Q*-function. Each *i*-th SU at location $l_{i}=\left\{x_{si},y_{si}\right\}$ detects the PU presence from the aforementioned test statistics.
- The SUs then span the angle range of $\left[-\phi_{0},\phi_{0}\right]$ to find the direction of the signal from the PU. To elaborate, once an *i*-th SU receives some power from a PU, then based on its bore-sight angle $\theta_{ik}$ in the *k*-th sector, it determines the direction of the signal $\phi_{ik}$ where $\phi_{ik}$ lies between $\left[-\phi_{0},\phi_{0}\right]$. This phenomena is further illustrated in Figure 2.
- Select the grid points with location $l_{a}=\left\{x_{a},y_{a}\right\}$ for each SU *i* and assign a value $v_{i}$ to each grid point, where $v_{i}=1$ if the grid angle is between $\left[-\phi_{0},\phi_{0}\right]$ or else $v_{i}=0$.
- Aggregate the RSS values form the *N* SUs and select the grids with highest RSS values. Two different scoring strategies are selected here as follows:
  (10) $$f\left(v_{i}\right)=\left\{\begin{array}{c} v_{i}\\ v_{i}\times p_{i} \end{array}\right..$$
  Based on the above scoring strategies, the total number of grid points *K* are selected for the PU location estimation.
- In the final step, a grid-based centroid localization method is used to estimate the PU location, which is given as:
  (11) $${\hat{l}}_{p}=\frac{\sum_{a=1}^{K}w_{a}l_{a}}{\sum_{a=1}^{K}w_{a}},$$
  where $w_{a}=\frac{p_{i}-p_{\min}}{p_{\max}-p_{\min}}$, $p_{min}=\min\left\{p_{i}\right\}$, and $p_{max}=\max\left\{p_{i}\right\}$.

## 5. Localization Error Analysis

In order to analyze the error of the proposed PU localization method, the error is defined as $e={\left[{\hat{x}}_{p}-x_{p},{\hat{y}}_{p}-y_{p}\right]}^{T}$, where ${\hat{x}}_{p}$ and ${\hat{y}}_{p}$ are the estimated coordinates of the PU. The estimated two-dimensional coordinates of PU defined in (11) can be written as: ${\hat{x}}_{p}=\frac{\sum_{i=1}^{K}w_{i}x_{i}}{\sum_{i=1}^{K}w_{i}},$ and ${\hat{y}}_{p}=\frac{\sum_{j=1}^{K}w_{j}y_{j}}{\sum_{j=1}^{K}w_{j}},$ respectively. The squared error for the two-dimensional localization is defined as:

(12) $$\begin{array}{ccc} e^{2} & = & e_{x}^{2}+e_{y}^{2}\\ & = & {\left(\frac{\sum_{i=1}^{K}w_{i}\left(x_{i}-x_{p}\right)}{\sum_{i=1}^{K}w_{i}}\right)}^{2}+{\left(\frac{\sum_{j=1}^{K}w_{j}\left(y_{j}-y_{p}\right)}{\sum_{j=1}^{K}w_{j}}\right)}^{2}\\ & = & \frac{\sum_{i=1}^{K}\sum_{j=1}^{K}w_{i}w_{j}c_{ij}}{\sum_{i=1}^{K}\sum_{j=1}^{K}w_{i}w_{j}}, \end{array}$$

where $c_{ij}=\left(x_{i}-x_{p}\right)\left(x_{j}-x_{p}\right)+\left(y_{i}-y_{p}\right)\left(y_{j}-y_{p}\right)$. Consider that ${\overset{´}{x}}_{i}=\left(x_{i}-x_{p}\right)$ and ${\overset{´}{y}}_{i}=\left(y_{i}-y_{p}\right)$, then $c_{ij}$ is defined as $c_{ij}={\overset{´}{x}}_{i}{\overset{´}{x}}_{j}+{\overset{´}{y}}_{i}{\overset{´}{y}}_{j}$. Arranging the values of $c_{ij}$ in matrix form as $C=\overset{´}{x}{\overset{´}{x}}^{T}+\overset{´}{y}{\overset{´}{y}}^{T}$, where the dimensions of $\overset{´}{x}$ and $\overset{´}{y}$ is K×1. Now expressing the squared localization error in (12) in compact form as $e^{2}=\left(\frac{WCW^{T}}{W1W^{T}}\right)$, where $W∼N(\mu ,\sigma_{s}^{2}I)$ and 1 is the matrix of ones. Vector μ represents the mean values of W with elements $\mu_{i}=E\left[p_{i}\right]-p_{min}$, $\sigma_{s}^{2}$ is the variance, and I is the identity matrix. The mean square error can be defined as $E\left[e^{2}\right]=E\left[\frac{WCW^{T}}{W1W^{T}}\right]$, where E[·] is the expectation operator. The expression for the expectation is extracted by using the moment generating function (MGF), which is given as

(13) $$E\left[\frac{WCW^{T}}{W1W^{T}}\right]=\int_{0}^{\infty }\varphi \left(0,-t\right)\left(\mathrm{Tr}\left(A\right)+{\overset{´}{\mu }}^{T}A\overset{´}{\mu }\right)dt,$$

where Tr(·) is the trace operator and φ(0,−t) is the combined MGF of $A=Q^{T}CQ$ and $\overset{´}{\mu }=Q^{T}\mu$. Note that matrix Q is of the dimension K×K and satisfies the condition $Q^{T}Q=\frac{1}{\left(I+2t1\right)}=D\left(t\right)$. Now, the MGF function can be expanded as:

(14) $$\begin{array}{cc} \varphi (0,-t)= & \frac{1}{\sqrt{\left|(I+2t1)\right|}}\\ & \times \exp^{\left(\frac{1}{2}\left(\mu^{T}{\left(I+2t1\right)}^{-1}\mu -\mu^{T}\mu \right)\right)}, \end{array}$$

where |·| represents the determinant. Simplifying (14), we get:

(15) $$\begin{array}{cc} \varphi (0,-t)= & \frac{1}{\sqrt{\left|(I+2t1)\right|}}\\ & \times \exp^{\left(\frac{\mu^{T}{\left(I+2t1\right)}^{-1}\mu }{2}-\frac{\mu^{T}\mu }{2}\right)} \end{array}$$

Note that |(I+2t1)|=1+2Kt, which yields

(16) $$\varphi \left(0,-t\right)=\frac{1}{\sqrt{1+2Kt}}\exp^{\left(\frac{\mu^{T}D\left(t\right)\mu }{2}-\frac{\mu^{T}\mu }{2}\right)}.$$

The exponential terms can be expanded as follows:

(17) $$\begin{array}{ccc} \frac{\mu^{T}D\left(t\right)\mu }{2}-\frac{\mu^{T}\mu }{2} & = & \frac{\mu^{T}\mu }{2}-\frac{t\mu^{T}1\mu }{1+2Kt}-\frac{\mu^{T}\mu }{2}\\ & = & -\frac{t\mu^{T}1\mu }{1+2Kt}. \end{array}$$

Substituting (17) in (16) yields the expression for joint MGF—that is:

(18) $$\varphi \left(0,-t\right)=\frac{1}{\sqrt{1+2Kt}}\exp^{\left(-\frac{t\mu^{T}1\mu }{1+2Kt}\right)}.$$

The expressions for Tr(A) and ${\overset{´}{\mu }}^{T}A\overset{´}{\mu }$ in (13) are derived as follows:

(19) $$\begin{array}{ccc} \mathrm{Tr}(A) & = & \mathrm{Tr}\left(Q^{T}CQ\right)=\mathrm{Tr}\left(CQQ^{T}\right)=\mathrm{Tr}\left(CD\left(t\right)\right)\\ & = & \mathrm{Tr}\left(C\right)-\frac{2t\mathrm{Tr}(C1)}{1+2Kt} \end{array}$$

and

(20) $$\begin{array}{ccc} {\overset{´}{\mu }}^{T}A\overset{´}{\mu } & = & \mu^{T}QAQ^{T}\mu =\mu^{T}QQ^{T}CQQ^{T}\mu \\ & = & \mu^{T}Z\left(t\right)\mu \end{array}$$

where Z(t)=D(t)CD(t). By expanding Z(t), we get:

(21) $$Z\left(t\right)=C-\frac{2tC1}{1+2Nt}+{\left(\frac{2t}{1+2Nt}\right)}^{2}1C1.$$

Substituting (21) in the left-hand side of (20) yields:

(22) $$\begin{array}{ccc} \mu^{T}Z\left(t\right)\mu & = & \mu^{T}C\mu -\frac{2t}{1+2Kt}\mu^{T}C1\mu \\ & & -\frac{2t}{1+2Kt}\mu^{T}1C\mu \\ & & +{\left(\frac{2t}{1+2Kt}\right)}^{2}\mu^{T}1C1\mu . \end{array}$$

Combining the expressions for Tr(A) and ${\overset{´}{\mu }}^{T}A\overset{´}{\mu }$, we get

(23) $$\begin{array}{ccc} \mathrm{Tr}\left(A\right)+{\overset{´}{\mu }}^{T}A\overset{´}{\mu } & = & \mathrm{Tr}\left(C\right)+\mu^{T}C\mu \\ & & -\frac{2t\mathrm{Tr}(C1)}{1+2Kt}-\frac{4t\mu^{T}A1\mu }{1+2Kt}\\ & & +{\left(\frac{2t}{1+2Kt}\right)}^{2}\mu^{T}1C1\mu . \end{array}$$

By substituting (18) and (23) in (13), we obtain the final expression for the mean square error as follows:

(24) $$\begin{array}{ccc} E[e^{2}] & = & \int_{0}^{\infty }\frac{1}{\sqrt{1+2Kt}}\exp^{\left(-\frac{t\mu^{T}1\mu }{1+2Kt}\right)}\\ & & \left(\mathrm{Tr}\left(C\right)+\mu^{T}C\mu -\frac{2t\mathrm{Tr}(C1)}{1+2Kt}-\frac{4t\mu^{T}A1\mu }{1+2Kt}\right.\\ & & \left.+{\left(\frac{2t}{1+2Kt}\right)}^{2}\mu^{T}1C1\mu \right)dt. \end{array}$$

## 6. Numerical Results

**Figure 3:**
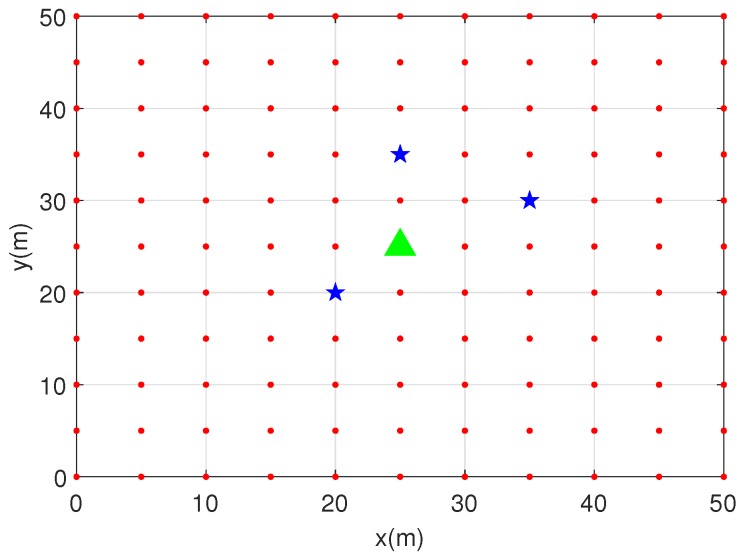
CRN setup in a 50 × 50 square area, where the area is divided into 50 × 50 grids with the resolution of 5 m. Location of PU is [25 m, 25 m], and three SUs are selected.

In this section, we perform numerous simulations to evaluate the performance of the proposed localization method. A 50 m × 50 m square area is considered where the area is divided into 11 × 11 grids with the resolution of 5 m, as shown in Figure 3. The location of PU is assumed to be [25 m, 25 m], and a set of SUs are randomly selected within the area, following uniform distribution. Here, we assume a 5G directional CRN setup, where the SUs are equipped with multiple directive antennas and are able to detect the PU signal. The SUs are equipped with three sector antennas with random orientation and beamwidth of 2π/3. Transmit power of the PU is set to 20 dBW, with path-loss exponents of 3 and 5, respectively. The shadowing effect is considered to be $\sigma_{s}=0$ dB and $\sigma_{s}=10$ dB, respectively. The performance metric for the proposed localization method is considered to be the root mean square error (RMSE), that is, $\mathrm{RMSE}=\sqrt{\frac{\sum_{i=1}^{R}{\left({\hat{x}}_{pi}-x_{p}\right)}^{2}+\sum_{i=1}^{R}{\left({\hat{y}}_{pi}-y_{p}\right)}^{2}}{R}},$ where *R* is the total number of estimated PU locations and ${\hat{x}}_{pi}$, ${\hat{y}}_{pi}$ is the two-dimension location of the PU for a single realization.

**Figure 4:**
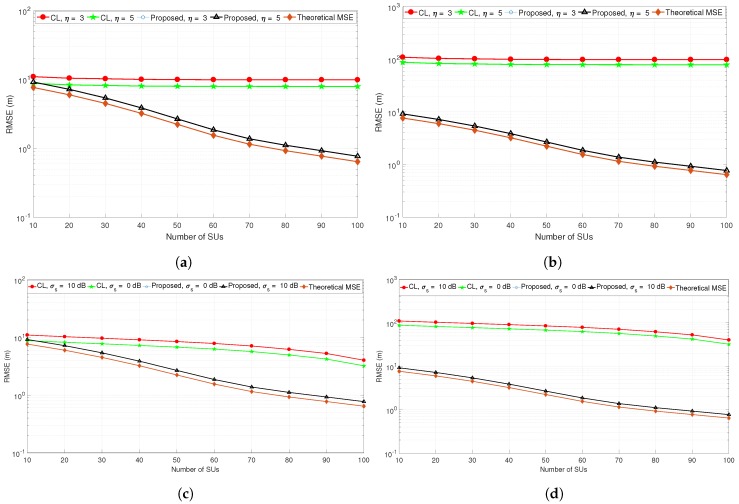
(**a**) RMSE when PU is located at [25 m, 25 m]; (**b**) RMSE when PU is located at [40 m, 10 m]; (**c**) impact of η and $\sigma_{s}$ when PU is located at [25 m, 25 m]; (**d**) impact of η and $\sigma_{s}$ when PU is located at [40 m, 10 m].

Figure 4a,b compares the performance of the proposed localization method with the centroid localization technique when the number of SUs are increased from 10 to 100. Note that increasing the number of SUs improves the RMSE performance up to a certain level, after which the RMSE remains stable and the performance improvement is not obvious. Surprisingly, the performance of the proposed PU localization method in Figure 4a,b follows the same pattern, which means that the proposed method is robust to the scenario settings, while the performance of the centroid localization method is highly affected by changing the scenario. Specifically, when the PU location is moved from the center to the edge, the performance of centroid localization is highly degraded due to the center-biased nature of these methods. Even though an increase in the number of SUs will not help to reduce the localization error, the proposed PU localization method is robust to the PU location as long as a sufficient number of SUs is available. The effect of the path-loss exponent has also been evaluated in Figure 4a,b, where the RMSE for centroid localization is large for a small value of η. Furthermore, the effect of shadowing $\sigma_{s}$ was tested in Figure 4c,d for two different PU locations. Note that the proposed PU localization method is more robust to the shadowing effects as compared to the centroid localization methods.

**Figure 5:**
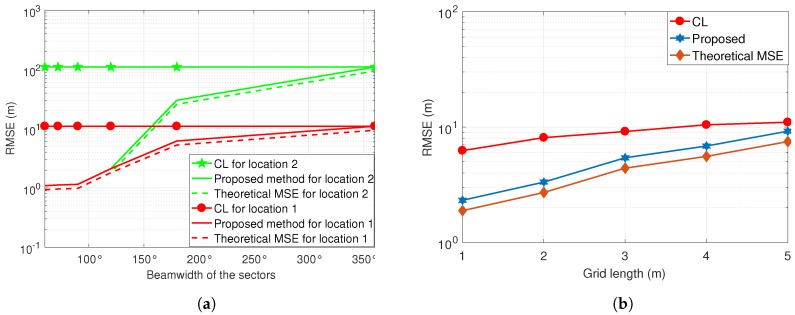
(**a**) Impact of beam width on RMSE; (**b**) impact of grid size on RMSE.

In Figure 5a, the impact of SU sectors is investigated for two different PU locations. Figure 5a shows that reducing the sector size improves the localization accuracy, because the closer grid points to the PU have been selected for final position estimation. Additionally, the impact of PU location is also shown in Figure 5a, where the location of the PU in the center (at [25 m, 25 m]) of the network is estimated with better accuracy as compared to the PU located at [40 m, 10 m]. Furthermore, Figure 5a shows that increasing the sector beamwidth up to 2π results in the conventional CL technique.

Since the grid-based localization algorithms strongly depend on the size of the grid, we evaluated the performance of the proposed method with five different grid sizes, as shown in Figure 5b. The simulation parameters for the results in Figure 5b consists of a PU located at [25 m, 25 m], where number of SUs are 10, η=3, and $\sigma_{s}=10$ dB. Figure 5b shows that the smaller grid size results in better localization accuracy, because closer grid points are selected to localize the PU. However, reducing the size of the grid can significantly increase the complexity of the network. Also, it is shown in Figure 5b that the proposed localization method outperforms the CL method due to the use of directional antennas to select the grids near to the PU.

## 7. Conclusions

In this paper, we have proposed a grid-based PU localization method where the SUs are equipped with the sectorized antennas. Different scoring functions were introduced, where the location of the PU was estimated by the grids with highest score function. Moreover, for the theoretical analysis, the expression for mean square error of the proposed method was derived. Furthermore, the RMSE of the proposed localization method was compared with the CL method under different simulation setups, where the results show that the proposed method has better localization accuracy and is robust to the scenario settings.

## References

[1] Axell E., Leus G., Larsson E., Poor H. (2012). Spectrum Sensing for Cognitive Radio: State-of-the-Art and Recent Advances. IEEE Signal Process. Mag..

[2] Ma J., Li G.Y., Juang B.H. (2009). Signal Processing in Cognitive Radio. Proc. IEEE.

[3] Lu L., Zhou X.W., Onunkwo U., Li G.Y. (2012). Ten Years of Cognitive Radio Technology. EURASIP J. Wirel. Commun. Netw..

[4] Mitola J., Maguire G. (1999). Cognitive radio: Making software radios more personal. IEEE Pers. Commun..

[5] Nam H., Ghorbel M.B., Alouini M.S. Location-Based Resource Allocation for OFDMA Cognitive Radio Systems. Proceedings of the IEEE International Symposium on Personal Indoor and Mobile Radio Communications, (PIMRC).

[6] Chen R., Park J.M., Reed J.H. (2008). Defense against primary user emulation attacks in cognitive radio networks. IEEE J. Sel. Areas Commun..

[7] Anand S., Jin Z., Subbalakshmi K. An analytical model for primary user emulation attacks in cognitive radio networks. Proceedings of the 3rd IEEE Symposium on New Frontiers in Dynamic Spectrum Access Networks.

[8] Chin W.L., Tseng C.L., Tsai C.S., Kao W.C., Kao C.W. Channel-based detection of primary user emulation attacks in cognitive radios. Proceedings of the IEEE 75th Vehicular Technology Conference (VTC Spring).

[9] Le T.N., Chin W.L., Lin Y.H. Non-cooperative and cooperative PUEA detection using physical layer in mobile OFDM-based cognitive radio networks. Proceedings of the International Conference on Computing, Networking and Communications (ICNC).

[10] Li Y., Ma X., Wang M., Chen H., Xie L., Panigrahi B.K., Trivedi M.C., Mishra K.K., Tiwari S., Singh P.K. (2019). Detecting Primary User Emulation Attack Based on Multipath Delay in Cognitive Radio Network. Smart Innovations in Communication and Computational Sciences.

[11] Ghanem W.R., Shokair M., Desouky M.I. An improved primary user emulation attack detection in cognitive radio networks based on firefly optimization algorithm. Proceedings of the 33rd National Radio Science Conference (NRSC).

[12] Fihri W.F., Ghazi H.E., Kaabouch N., Majd B.A.E. Bayesian decision model with trilateration for primary user emulation attack localization in cognitive radio networks. Proceedings of the International Symposium on Networks, Computers and Communications (ISNCC).

[13] Fihri W.F., Arjoune Y., Ghazi H.E., Kaabouch N., Majd B.A.E. A particle swarm optimization based algorithm for primary user emulation attack detection. Proceedings of the 2018 IEEE 8th Annual Computing and Communication Workshop and Conference (CCWC).

[14] El Mrabet Z., Arjoune Y., El Ghazi H., Abou Al Majd B., Kaabouch N. (2018). Primary User Emulation Attacks: A Detection Technique Based on Kalman Filter. J. Sens. Actuator Netw..

[15] Saeed N., Nam H. (2015). Robust Multidimensional Scaling for Cognitive Radio Network Localization. IEEE Trans. Veh. Technol..

[16] Khan M.A., Saeed N., Ahmad A.W., Lee C. (2017). Location Awareness in 5G Networks Using RSS Measurements for Public Safety Applications. IEEE Access.

[17] Liu Y., Shi X., He S., Shi Z. (2017). Prospective Positioning Architecture and Technologies in 5G Networks. IEEE/ACM Trans. Netw..

[18] Zafari F., Gkelias A., Leung K.K. (2017). A Survey of Indoor Localization Systems and Technologies. arXiv.

[19] Saeed N., Nam H. (2016). Cluster Based Multidimensional Scaling for Irregular Cognitive Radio Networks Localization. IEEE Trans. Signal Process..

[20] Saeed N., Haris M., Haq M.I.U. Jointly locating the primary and secondary users in cognitive radio networks. Proceedings of the International Conference on Communication, Computing and Digital Systems (C-CODE).

[21] Koivisto M., Hakkarainen A., Costa M., Kela P., Leppanen K., Valkama M. (2017). High-Efficiency Device Positioning and Location-Aware Communications in Dense 5G Networks. IEEE Commun. Mag..

[22] Türkyilmaz O., Alagöz F., Gür G., Tugcu T. (2008). Environment-aware Location Estimation in Cellular Networks. EURASIP J. Adv. Signal Process..

[23] Bulusu N., Heidemann J., Estrin D. (2000). GPS-less low-cost outdoor localization for very small devices. IEEE Pers. Commun..

[24] Mariani A., Kandeepan S., Giorgetti A., Chiani M. Cooperative weighted centroid localization for cognitive radio networks. Proceedings of the 2012 International Symposium on Communications and Information Technologies (ISCIT).

[25] Nam H., Saeed N., Ben-Ghorbel M., Alouini M.S. (2015). Primary user localisation and uplink resource allocation in orthogonal frequency division multiple access cognitive radio systems. IET Commun..

[26] Saeed N., Nam H. (2017). Energy Efficient Localization Algorithm With Improved Accuracy in Cognitive Radio Networks. IEEE Commun. Lett..

[27] Magowe K., Kandeepan S. Cooperative blind localization of primary user in a cognitive radio environment. Proceedings of the 2014 8th International Conference on Signal Processing and Communication Systems (ICSPCS).

[28] Wang J., Chen J., Cabric D. (2013). Cramer-Rao Bounds for Joint RSS/DoA-Based Primary-User Localization in Cognitive Radio Networks. IEEE Trans. Wirel. Commun..

[29] Wang J., Chen J., Cabric D. (2013). Stansfield Localization Algorithm: Theoretical Analysis and Distributed Implementation. IEEE Wirel. Commun. Lett..

[30] Penna F., Cabric D. (2013). Cooperative DoA-only localization of primary users in cognitive radio networks. EURASIP J. Wirel. Commun. Netw..

[31] Buyukcorak S., Kurt G.K., Yongacoglu A. Received signal strength based localization in sectorized cellular networks. Proceedings of the 2016 23rd International Conference on Telecommunications (ICT).

[32] Huang L., Zhu G., Du X. (2013). Cognitive femtocell networks: An opportunistic spectrum access for future indoor wireless coverage. IEEE Trans. Wirel. Commun..

[33] Wang C.X., Haider F., Gao X., You X.H., Yang Y., Yuan D., Aggoune H.M., Haas H., Fletcher S., Hepsaydir E. (2014). Cellular architecture and key technologies for 5G wireless communication networks. IEEE Wirel. Commun. Mag..

[34] Digham F.F., Alouini M.S., Simon M.K. (2007). On the Energy Detection of Unknown Signals Over Fading Channels. IEEE Trans. Commun..

[35] Lehtomaki J.J., Juntti M., Saarnisaari H. (2005). CFAR strategies for channelized radiometer. IEEE Signal Process. Lett..

